# Mechanical Aids in the Treatment of Pocket Disease of the Alveolus*Dr. Farrar desires it to be distinctly understood that any dentist is at perfect liberty to make use of any of the devices which he has, either in this article or at any previous time, presented to the profession. He has never in any way profited, or desired to profit, pecuniarily, by the manufacture or sale of any dental implements which he has originated. They are his contribution to the general good of the profession.—Editor.

**Published:** 1885-10

**Authors:** J. N. Farrar


### Independent Practitioner.

Vol. VI. October, 1885. No. 10.

### Original Communications.

### MECHANICAL AIDS IN THE TREATMENT OF POCKET DISEASE OF THE ALVEOLUS.*

BY J. N. FARRAR, M. D., D. D. S.

Without entering into the subject of etiology, it may be broadly stated that in order to reach the highest possibilities by any plan of treatment of what is known as "pyorrhœa alveolaris," and what I will here call, without stopping to give reasons, *pocket disease of the alveolus*, as much depends upon the quality of treatment as the kind—upon thoroughness of the mechanical aspect as upon the kind of medicine. This, however, includes a degree of cleanliness that implies proper instruments as well as intelligence. Attention has not been sufficiently directed to the devising of instruments that will permit rapid thoroughness with the least injury to the tissues, and the least pain. Waste of time and needless torture have too often usurped the place of thoroughness, and mere puttering assumed that of careful skill.

In the construction of instruments, proper size is of as much importance as correct form. While variety of shape is essential in

* Dr. Farrar desires it to be distinctly understood that any dentist is at perfect liberty to make use of any of the devices which he has, either in this article or at any previous time, presented to the profession. He has never in any way profited, or desired to profit, pecuniarily, by the manufacture or sale of any dental implements which he has originated. They are his contribution to the general good of the profession.—Editor.

order to easily reach a locality, delicacy in structure is necessary to effectiveness, with the least pain and the least injury to the soft tissues. Small and delicate instruments permit the attainment of all that is desired; clumsy ones cannot. Different sizes, although of the same shape, may also be said to be sufficient to constitute practically as great a difference as different forms. A small bistoury in the hands of a tyro will delicately open tissues where a large scalpel in the hands of skill could only butcher. If many failures and a vast amount of injury are attributable to a bungling use of the best of instruments, and the result of the use of colossal "tools" in the hands of skill cannot be much better, the results of a combination of the bungler and the bungling can be easily imagined.

While scrapers and cutting instruments should be made with this especial reference, syringes for washing the debris from the pockets should also be constructed with the view to equal thoroughness. Unlike those in present use, which more resemble hypodermic syringes than anything else, holding only a few drops, they should be capable of holding sufficient water to flood out the pockets. A few drops of water will wet, but sufficient quantity is requisite to thorough cleansing.

Although but few properly made instruments for this purpose are to be found in the shops, several operators who have felt the need have devised for their own use several varieties, some of which should be made public for the good of the profession, for as long as this pocket disease is treated with no more appropriate instruments than the big, old-fashioned "tartar scrapers," and the teaspoon syringes, the advance along the line of the profession in this matter will be irregular and unsatisfactory.

In the hope of stimulating greater interest in others to make their own, I will briefly explain a set of instruments that I have from time to time devised by scribe rule (trial fit) for my own use, which enables me to operate more rapidly and more thoroughly, with less injury and less pain, than by any instruments which I have been able to find in the shops. An approximate idea of these may be obtained by the preceding diagram, but it should be remembered that instruments having such varied curvatures in shank and blade cannot be accurately shown. Slight variation of shape, especially in the shank, is sufficient to make the difference between practicable and impracticable.

When a location of the di ease was found that could not be easily reached with anything that I had, I selected a rod of soft steel from a lot partially prepared for the purpose, and bent, hammered, filed and fitted it on the spot, and, after using it for a week or two to see that it was all right, it was tempered for permanent use. In this way a set of about thirty instruments came into existence. While some are more generally useful than others, yet for the purpose intended all are valuable to me, but perhaps a dozen might be selected that would suffice.

In this set, instead of the smallest peach-leaf hoe-scrapers being five-eighths of an inch in length, as usually found in the shops, the largest is only three-eighths of an inch, and they range down to one thirty-second of an inch in length, while the pocket-hoes, such as Nos. 13 and 14, are very much less. Another peculiarity of these peach-leaf blades lies in their points being rounded, some of which also have only one cutting edge, in order to avoid deepening of the pockets or lacerating the soft tissues while scraping the roots. This is important, for while bleeding to a limited extent is beneficial, unnecessary injury of these tissues is injurious.

Fig. 2 approximately illustrates one or two views of the shanks and blades of twenty of the set of cutting and scraping instruments referred to, but as Nos. 6, 11, 17, and 20 are in pairs, and only one of each is shown in the diagram, they practically illustrate twenty-four, as follows :

No. 1—Straight shank, curved blade hoe, for cervical work.

No. 2—Two views of one of a pair of molar cervical peach-leaf-shaped hoes, with a bowed shank to hold the cheek away.

No. 3—A chisel for use between the lower front teeth (from the inside).

No. 4—A thin chisel for use between teeth from the outside.

No. 5—Hoe scaler for side teeth.

No. 6—Two views of a root scraper, with curved shank for a pushing movement.

No. 7—Straight shank, root push-scraper.

No. 8—Four sizes of peach-leaf hoe scrapers, double edge.

No. 9—Two views of curved shank, peach-leaf hoe (delicate).

No. 10—Double bent shank, peach-leaf hoe, (delicate).

No. 11—Double bent shank, side, right and left, peach-leaf hoe, (delicate ; two views).

No. 12—Root hoe-scraper with curved shank, side blade, (two views).

No. 13—Root hoe-scrapers, straight shank (should be more delicate than here shown).

Nos. 14, 15, and 16—Different sizes of single curved shank, root hoe-scrapers.

Nos. 17 and 18—Different views of triple curved shank scrapers, with side blade for approximal surfaces.

Nos. 19 and 20—Different views of single curved shank, cervical hoes, with one sharp edge only.

While the cut shows the general forms of the instruments, the size and length of the shanks may be varied to advantage, but they should all be as delicate as is possibly consistent with firm rigidity, and tempered very hard. Any elasticity is a disadvantage. The handles may be file-cut steel, but are better if made of wood.

All root hoes should be very small, about the size of No. 14, or even less. The plain views shown, Nos. 12–13, are too large.

After the hard deposits have been detached from the roots, or after cutting loose necrotic tissue (when present), it is necessary, in order to effect the most rapid cure, that the debris be thoroughly washed away, leaving no particles to be "ulcerated out," as is too often the case. This requires water in sufficient quantity to flood out the pockets with a vigorous stream, long continued, which implies a syringe of considerable size. I make use of four syringes for this purpose, with capacities varying from two to four ounces, which I had made from my own designs. (See Fig. 1.)

While the nozzle is of the same form as those used on the Farrar Alveolar Abscess Syringe (Fig. 3), the points are somewhat larger than the "proboscis size;" indeed, several sizes are necessary to meet all cases. While one end of the body of the nozzle is screw-threaded to firmly fix it to the syringe, the detachable thumb-hub to which it is attached has a smooth hole through it, which fits a tapering stub, enabling it to be easily and quickly removed and readjusted when charging the syringe with water. By way of variety, one of my larger syringes has a little hollow thumb-wheel stop-cock set in a fixed hub, but this is not as convenient as the other.

As these delicate points of the nozzles would be easily broken by the weight of such large syringes, finger-hooks are made to project from an annular ring around the head of the syringe, to enable it to be hung upon the edge of a tumbler, as shown in the figure. These hooks are also useful in grasping and holding the syringe firmly while in use.

Fig. 4 illustrates a rubber tubing, sometimes useful in painful cases, to interpose between the syringe and the diseased tissue, to cut off the jar unavoidable in driving the water out. This, however, is seldom necessary.

Without enlarging upon the subject by stating reasons, I will conclude by saying (as stated in a former paper published in the *Missouri Dental Journal*, June 1879, page 305), after thoroughly washing the pockets with tepid water, and if there is no necrosis, I inject them with equal parts of wood creosote and alcohol from a Farrar Alveolar Abscess Syringe (Fig. 3) ; but if necrotic tissue is present, I use chloride of zinc, just short of escharotic strength.

